# The effectiveness of hormonal treatment to improve reproductive outcomes in normogonadotropic men with abnormal semen parameters: results of 2 linked systematic reviews

**DOI:** 10.1210/clinem/dgag185

**Published:** 2026-04-24

**Authors:** Kristin Konnyu, Mari Imamura, Jemma Hudson, James Swingler, Paul Manson, Siladitya Bhattacharya, Channa N Jayasena, Miriam Brazzelli

**Affiliations:** Aberdeen Centre for Evaluation (ACE), University of Aberdeen, Aberdeen AB25 2ZD, UK; Aberdeen Centre for Evaluation (ACE), University of Aberdeen, Aberdeen AB25 2ZD, UK; Aberdeen Centre for Evaluation (ACE), University of Aberdeen, Aberdeen AB25 2ZD, UK; Aberdeen Centre for Evaluation (ACE), University of Aberdeen, Aberdeen AB25 2ZD, UK; Aberdeen Centre for Evaluation (ACE), University of Aberdeen, Aberdeen AB25 2ZD, UK; School of Medicine, Medical Sciences, and Nutrition, University of Aberdeen, Aberdeen AB25 2ZD, UK; Department of Metabolism, Digestion and Reproduction, Faculty of Medicine, Imperial College London, London W12 0NN, UK; Aberdeen Centre for Evaluation (ACE), University of Aberdeen, Aberdeen AB25 2ZD, UK

**Keywords:** male infertility, abnormal semen parameters, normogonadotropic men, hormonal treatment, systematic review, meta-analysis

## Abstract

**Context:**

Growing numbers of international guidelines recommend hormonal treatment for normogonadotropic men with abnormal semen parameters, despite uncertain effects on fertility outcomes.

**Objective:**

Evaluate the efficacy of hormonal treatment, compared with placebo or no treatment, in normogonadotropic men with abnormal semen parameters.

**Data Sources:**

We searched MEDLINE, Embase, the Cochrane Database of Systematic Reviews, and Cochrane Central Register of Controlled Trials to February 2025.

**Study Selection:**

Two linked reviews were conducted simultaneously. Review 1 included randomized and nonrandomized controlled studies evaluating hormonal treatments in normogonadotropic men with abnormal semen parameters. Review 2 included the same treatments in men undergoing assisted reproduction. All citations were double-screened.

**Data Extraction:**

Primary outcomes were changes in semen quality (in Review 1) and pregnancy (in Review 2). Risk of bias of the included studies was assessed using the Cochrane tool and ROBUST-RCT. Random-effects meta-analyses were performed.

**Data Synthesis:**

Nine studies (735 men) were included. Follicle-stimulating hormone showed modest improvements in sperm count (mean difference [MD] 11.0; 95% CI, 7.2-14.8), concentration (MD 5.6; 95% CI, 1.9-9.3), and motility (MD 3.4; 95% CI, 0.7-6.0) but not morphology (MD 4.7; 95% CI, −0.8 to 10.3). Studies of combined human chorionic and menopausal gonadotropins, as well as tamoxifen, showed no benefit. Two small studies reported nonsignificant increases in pregnancy with FSH. No eligible studies evaluated aromatase inhibitors or clomiphene.

**Conclusion:**

We cautiously report potential for selective use of FSH in normogonadotropic men. However, the expected benefits are modest and short-term, with uncertain translation to pregnancy or live birth.

Global estimates suggest that male factor infertility accounts for approximately one half of all cases of involuntary childlessness in couples (1, 2). Among these, 30% to 40% of men exhibit normal findings on physical examination and hormonal laboratory tests, with no identifiable pathological cause to explain impaired semen quantity and quality. This condition has historically been identified as idiopathic male infertility (1). Idiopathic male infertility is characterized by 3 key sperm anomalies: low sperm count (oligozoospermia), poor sperm motility (asthenozoospermia), and abnormal sperm morphology (teratozoospermia). These anomalies often, but not always, occur together, a condition referred to as oligo-astheno-teratozoospermia (OAT) syndrome (1). With its unknown etiology, idiopathic male infertility remains challenging to treat.

Hormonal therapies are established and highly effective treatments stimulating spermatogenesis and enabling fertility in men with hypogonadotropic hypogonadism (3-5). However, the use of hormonal therapy for men with normogonadotropic idiopathic infertility (eg, OAT syndrome in the presence of normal hormone levels), remains unproven (4, 6). Proposed hormonal treatments for men with normogonadotropic idiopathic infertility include gonadotropin therapies, selective estrogen receptor modulators (SERMs), and aromatase inhibitors.

Clinical guidelines offer mixed recommendations for hormonal treatments for men with idiopathic infertility, and if treatment is recommended, it is with low confidence. For example, NICE Clinical Guideline 156 (7) recommends offering treatment with gonadotropin drugs to men with low levels of gonadotropins but states there is insufficient evidence to support the use of gonadotropins or other fertility drugs to improve fertility in cases of sperm dysfunction with no identifiable cause. The American Urological Association guidelines (8) suggest that for men with idiopathic infertility, clinicians may consider treatment using FSH to improve sperm concentration, pregnancy rate, and live birth rate, but indicate that in patients with nonobstructive azoospermia, clinicians may highlight the limited data supporting pharmacologic manipulation with selective estrogen receptor modulators, aromatase inhibitors, and gonadotropins before surgical intervention. The European Association of Urology Guidelines 2014 (9) provide a weak recommendation to use FSH treatment in men with idiopathic oligozoospermia and FSH values within the normal range, but recommend against using high-dose FSH because of insufficient evidence. With respect to other treatments, they state there is no conclusive evidence supporting the beneficial effects of SERMs or aromatase inhibitors. The European Academy of Andrology guideline for the management of OAT (10) suggests that treatment with FSH may be considered for selected men from couples experiencing infertility—specifically, normogonadotropic men with idiopathic oligozoospermia or OAT—although the quality of evidence underpinning this recommendation is low. This guideline also states that, based on current evidence, no definitive recommendation can be made either for or against the use of antioxidants, antiestrogens (such as tamoxifen or clomiphene), or aromatase inhibitors.

Given the current uncertainty, we performed a systematic review to assess the evidence regarding the impact of hormonal treatments for men with idiopathic infertility on semen parameters and pregnancy outcomes. Specifically, we aimed to answer 2 interconnected review questions:

1. What is the effectiveness of hormonal treatment in improving semen parameters in men who are normogonadotrophic and have abnormal semen parameters? (Review 1)
2. In couples undergoing assisted reproduction techniques—such as intrauterine insemination, in vitro fertilization (IVF), or intracytoplasmic sperm injection (ICSI)—what is the effect of hormonal treatment administered to normogonadotrophic male partners with abnormal semen parameters on pregnancy outcomes? (Review 2)

## Materials and methods

The 2 systematic reviews were conducted according to a prespecified protocol that was developed with input from scientific experts at Merck Serono KGaA as well as from our clinical experts (S.B., C.J.). The protocol was used internally to ensure methodological consistency; however, preregistration was not undertaken because the review was initiated under time-limited project constraints. The reviews were conducted in accordance with the methodological recommendations outlined in the *Cochrane Handbook for Systematic Reviews of Interventions* (11) and are reported according to the *Preferred Reporting Items for Systematic Review and Meta-Analysis Protocols* 2020 guidelines (12) (Appendix A) (13). Any changes to the protocol and their rationale are detailed in Appendix B (13).

### Data sources and searches

We conducted comprehensive literature searches in MEDLINE (via Ovid), Embase, the Cochrane Database of Systematic Reviews, and the Cochrane Central Register of Controlled Trials. The date of the last search was February 17, 2025. The search strategies were designed to capture the main population, intervention, comparator, outcome (PICO) elements of the operationalized review questions (Appendix C) (13) including database index terms and free text for abnormal sperm parameters, hormonal treatments, and assisted reproduction. There were no restrictions on outcome measures, study type, or language at the search stage. The search strategies for Ovid MEDLINE for both systematic reviews can be found in Appendix D (13). All identified references were exported to Endnote for recording and deduplication before being uploaded to the systematic review software, Nested Knowledge (https://nested-knowledge.com/). The reference lists of all included articles were screened for additional relevant studies.

### Study selection

All identified citations were independently double-screened. Initially this was done by a team of two human reviewers (K.K., M.I.). After a training phase of 100 records using the Nested Knowledge automated software, screening was conducted by a human reviewer in conjunction with a robot reviewer (K.K. or M.I.). Any conflicts arising during the human-robot screening phase were reviewed by a second human reviewer. All potentially relevant studies were retrieved and evaluated independently by the same 2 human reviewers. Discrepancies were resolved through discussion between the 2 reviewers, or, if needed, with the wider research team.

For Review 1, we included studies involving men with normal gonadotrophin levels, defined in collaboration with our expert partners as men with FSH and LH levels ≤10 IU/L and abnormal semen parameters according to either the World Health Organization (WHO) criteria (14) or the definitions provided by the study authors. For Review 2, we included studies involving couples undergoing assisted reproduction techniques, such as intrauterine insemination, IVF, and ICSI. In these studies, the male partner met the same criteria as in Review 1, namely, men with normal FSH and LH levels but with abnormal semen parameters.

For both reviews, we included comparative studies evaluating 1 or more of hormonal treatments of interest. These treatments included gonadotrophins (eg, recombinant FSH [rhFSH], urinary FSH [uFSH] including its highly purified form [uFSH-HP], recombinant LH, human chorionic gonadotrophin [hCG] including its highly purified form, human menopausal gonadotropin [hMG] including its highly purified form), SERMs (eg, clomiphene), and aromatase inhibitors (eg, letrozole, anastrozole). Studies were included if the comparator group received either no treatment or placebo.

In Review 1, our primary outcomes of interest were changes in semen parameters: sperm count, motility, and morphology. In Review 2, our primary outcome of interest was clinical or ongoing pregnancy. Secondary outcomes included live births, miscarriages, oocyte fertilization rate, number of embryos with good quality, embryo blastulation rate, and embryo implantation rate.

We included randomized controlled trials (RCTs) and nonrandomized controlled studies (NRCS) such as controlled clinical trials or cohort studies with a control group.

We excluded uncontrolled studies and commentaries (eg, case-control studies, single cohorts, case series, case reports, expert opinions, editorials, narrative reviews). Although systematic reviews were excluded, we screened their references for potentially relevant studies. Similarly, we excluded conference abstracts becauseof limited details reported but used them to identify additional relevant studies when possible.

### Data extraction and quality assessment

Data extraction, including study characteristics, baseline data, treatment details, and study outcomes, was initially conducted by 1 reviewer using the Nested Knowledge software and then exported to an Excel sheet on OneDrive, where a second reviewer verified the data for accuracy. In line with the Cochrane original Risk of Bias tool (15) and the more recent ROBUST-RCT tool (16), the methodological quality of the included studies was assessed based on 6 key domains: (1) random sequence generation; (2) allocation concealment; blinding of (3) participants, (4) health care providers, and (5) outcome assessors; and 6) missing outcome data. Risk of bias assessment was performed independently by 2 reviewers using a shared Excel file hosted on OneDrive.

### Data synthesis and analysis

We provided a description of the included studies based on their key clinical and methodological characteristics. Continuous outcomes were reported as mean differences (MD) with 95% CIs, whereas dichotomous outcomes were reported as odds ratios (OR), with corresponding 95% CIs. Where sufficient and comparable data were available, meta-analyses were conducted using random-effects models with the restricted maximum likelihood estimator and Hartung-Knapp-Sidik-Jonkman variance correction to account for potential between-study heterogeneity (17, 18). Heterogeneity was assessed both clinically (based on clinical criteria) and statistically. Visual inspection of forest plots and the chi-squared (χ^2^) test, and the *I*^2^ statistic were used to evaluate variability among studies. A *P* < .10 for the χ^2^ test, or an *I*^2^ value >50% was considered indicative of substantial heterogeneity. For Review 1, a sensitivity analysis was performed by excluding studies that showed clear evidence of substantial heterogeneity. Subgroup analyses based on testosterone and testosterone/epitestosterone values were planned for Review 1; however, we were unable to conduct such analyses because of insufficient relevant subgroup data. We also carefully considered the face validity of reported standard errors and standard deviations; where necessary and based on the context and magnitude of the values, we assumed that values labeled as standard errors had been misreported and in fact represented standard deviations. In 1 study that reported sperm motility in categorical form, we combined the categories using a standard formula with an assumed correlation of 0.7 (19). For studies comparing different doses of FSH, treatment groups were combined using established statistical methods.

Results of RCTs and NRCS were analyzed separately. All statistical analyses were conducted using Stata statistical software (version 17). A 2-tailed *P*-value <.05 was considered statistically significant for all outcome measures. For outcomes that could not be pooled quantitatively, a narrative synthesis was undertaken.

## Results

### Results of the search

**Figure 1:**
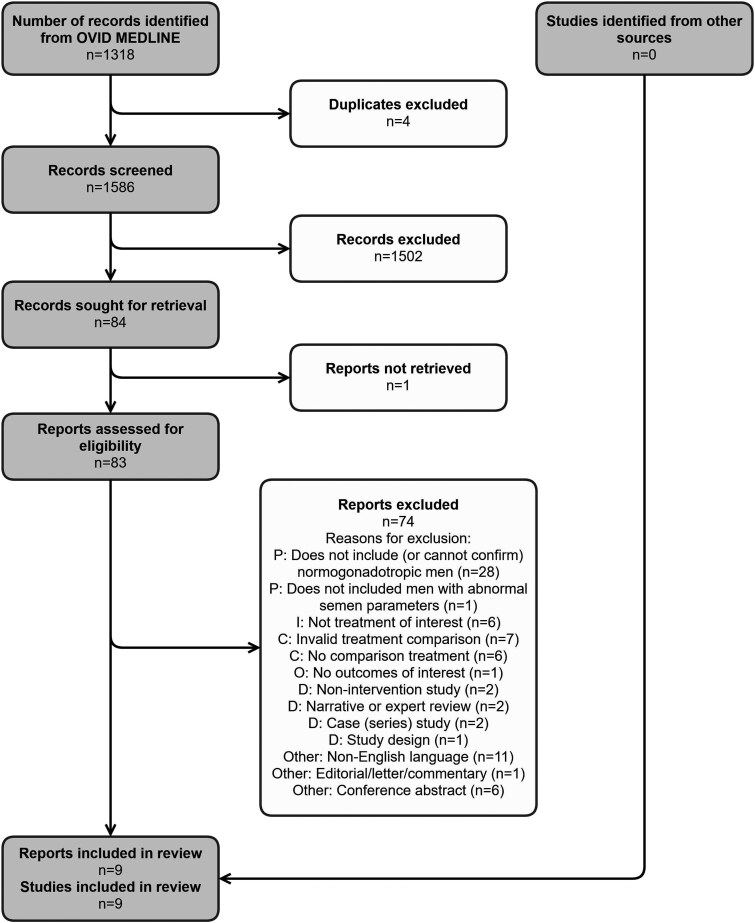
PRISMA flow diagram illustrating the study selection process (Review 1).

**Figure 2:**
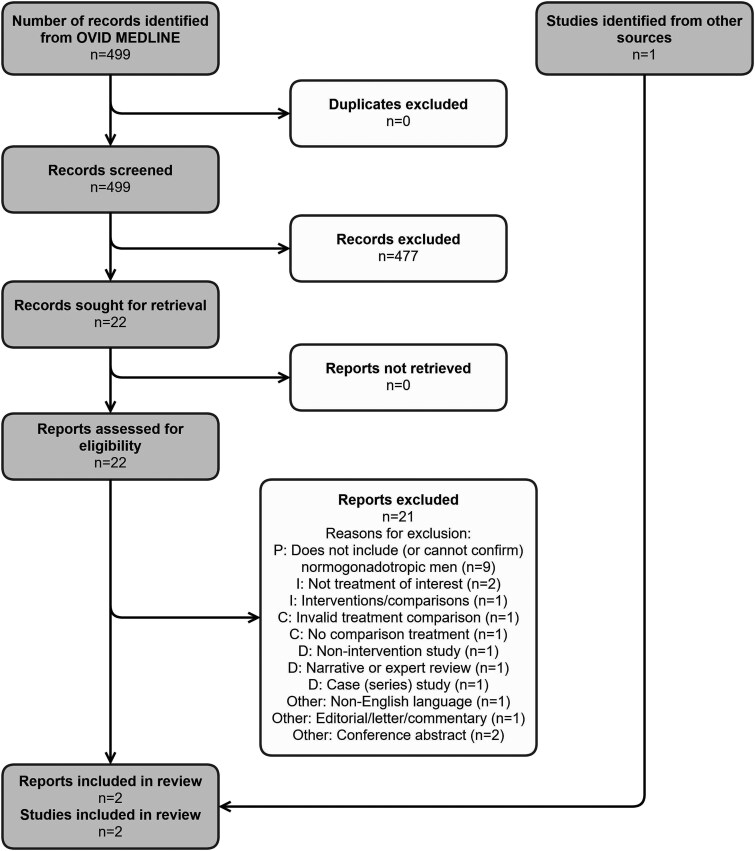
PRISMA flow diagram illustrating the study selection process (Review 2).

The literature searches identified 1318 citations for Review 1 and 499 citations for Review 2. After screening the titles and abstracts against the eligibility criteria, we retrieved and assessed 84 full-text articles for Review 1 and 22 full-text articles for Review 2. The final sample included 9 studies (reported across 9 publications) (20-28) for Review 1 and 2 studies (reported in 2 publications) (22, 26) for Review 2. Notably, the study by Paradisi et al (2014) is reported across 2 publications (24, 25) and the publication by Ding et al (2015) (26) comprises 2 separate trials. In total, 9 unique studies, comprising 735 men, were included across both reviews. Details of the screening process and reasons for exclusion are provided in Figs. 1 and 2.

### Included studies

Details on study design and patient characteristics are summarized in Tables 1 and 2, respectively. We assessed 8 RCTs (20-23, 26-28) involving a total of 675 patients and 1 NRCS with 60 patients (24) Of the 8 RCTs, 7 were parallel group randomized trials (20-23, 26, 27) and 1 was a randomized crossover study in which the same 16 patients received placebo or hormones and were evaluated before switching to the alternative arm (28). Seven of the 9 included studies were placebo-controlled (20, 23, 24, 26-28), whereas the remaining 2 used no-treatment control groups (21, 22). Eight studies evaluated gonadotropins (7 FSH, 1 combined hCG/hMG) (20-24, 26, 27) and 1 study a SERM (tamoxifen) (28). We did not identify any study that investigated the use of aromatase inhibitors. Four of the FSH studies were conducted in Italy by a single research group (20-23). One NRCS was led by another research group based in Italy (24) and 2 FSH trials, led by the same investigators, were conducted in China (26). The hCG/h|MG (27) and tamoxifen (28) trials were conducted in Germany and Canada, respectively. Studies were published between 1987 to 2015, though the specific dates of study conduct were generally poorly reported. Aside from 1 study (Ding et al) (26), which recruited patients from 3 hospitals, all studies were conducted in single infertility or university clinic settings. Participants were men between 28 and 36 years old on average, all meeting WHO criteria for abnormal semen parameters but presenting with normal baseline hormone levels. The duration of treatment and follow-up was typically 3 months, with some studies extended to 6 months. Although most studies disclosed the commercial source of the investigational treatments, none provided information regarding the nature of the relationship between the investigators and the sponsoring companies (eg, financial ties, potential intellectual conflicts of interest).

**Table 1:** Study characteristics of studies in Reviews 1 and 2

Author, Year, country, total N	Review	Design	Setting	Intervention	Comparator	Drug, company	Duration of treatment	Semen analysis guideline
**Gonadotropins (n = 8 studies)**								
***FSH (n* = *7 studies*** *)*								
**Foresta, 1998**, (20) Italy, N = 90	1	RCT	University clinic	75 IU uFSH-HP on alternate days	Placebo	Metrodin HP, Serono	3 months	WHO 1992
**Foresta, 2002**, (21) Italy, N = 45	1	RCT	University clinic	Arm 1: 50 IU rhFSH Arm 2: 100 IU rhFSH on alternate days	No treatment	Puregon, Organon	3 months	WHO 1999
**Foresta, 2005**, (22) Italy, N = 112	1, 2	RCT	University clinic	100 IU rhFSH on alternate days	No treatment	NR	3 months	NR
**Selice, 2011**, (23) Italy, N = 105	1	RCT	University clinic	150 IU rhFSH 3×/week	No treatment	Gonal F, Serono	3 months	WHO 1992
**Paradisi, 2014**, (24) Italy, N = 60	1	NRCS	O&G department	300 IU rhFSH on every other day	Placebo	Gonal-F, Merck-Serono	≥4 months	WHO 1999
**Ding, 2015a**, (26) China, N = 185	1, 2	RCT	3 hospitals	Arm 1: 50 IU rhFSH Arm 2: 100 IU rhFSH Arm 3: 200 IU rhFSH Arm 4: 300 IU rhFSH on alternate days	Placebo	Urofollitropin, Livzon Pharmaceutical	3 months	WHO (NR)*^a^*
**Ding, 2015b**, (26) China, N = 69	1, 2	RCT	3 hospitals	Arm 1: 300 IU rhFSH on alternate days	Placebo	Urofollitropin, Livzon Pharmaceutical	5 months	WHO (NR)*^a^*
***hCG + hMG (n* = *1 study)***								
**Knuth, 1987**, (27) Germany, N = 37	1	RCT	Infertility clinic	Combined: 2500 IU hCG (2×/week) + 150 IU hMG (3×/week)	Placebo	Pregnesin, Serono	3 months	WHO 1980
**SERMs (n = 1 study)**								
***Tamoxifen (n* = *1 study)***								
**AinMelk, 1987**, (28) Canada, N = 32	1	RCT crossover	Infertility clinic	20 mg tamoxifen citrate per day	Placebo	NR	6 months (each)	NR

There were no studies in the included sample that assessed the effectiveness of aromatase inhibitors for men with idiopathic infertility

Abbreviations: hCG, human chorionic gonadotropin; hMG, human menopausal gonadotropin; NR, not reported; NRCS, nonrandomized controlled trial; O&G, obstetrics and gynecology; RCT, randomized controlled trial; rhFSH, recombinant human FSH; SERM, selective estrogen receptor modulator; uFSH-HP, urinary human FSH highly purified.

^*a*^Based on year of publication, likely 2010 World Health Organization guidelines.

**Table 2:** Patient characteristics at baseline in Reviews 1 and 2

					Hormone parameters			Semen parameters			
Author, year, country, design	Arm	N analyzed	Age	Duration infertility (y)	LH (IU/mL)	FSH (IU/mL)	T (nmol/L)	Count (× 10^6^)	Concentration (× 10^6^/mL)	Morphology (% normal)	Motility*^a^* (%)
**Gonadotropins (n = 8 studies)**											
***FSH (n* = *7 studies*** *)*											
**Foresta, 1998**, (20) Italy, RCT	uFSH-HP 75	60	28 (5)*^b^*	NR*^b^*	3.1 (1.3)*^b^*	3.4 (1.1)*^b^*	15.8 (3.6)*^b^*	10.6 (3.4)	5.9 (3.1)	36.8 (6.1)	29.7 (5.1)
	Placebo	30	– (–)	– (–)	– (–)	– (–)	– (–)	8.1 (2.8)	4.8 (2.5)	33.4 (5.6)	31.8 (4.9)
**Foresta, 2002**, (21) Italy, RCT	rhFSH 50	15	33 (5)	NR	2.8 (1.2)*^b^*	4.1 (2.2)*^b^*	15.3 (8.3)*^b,c^*	NR	3.7 (1.8)	34.2 (10.1)	30.4 (7.5)
	rhFSH 100	15	34 (4)	NR				NR	5.1 (2.2)	37.8 (9.4)	35.5 (11.6)
	No tx	15	32 (4)	NR				NR	4.3 (2.1)	35.6 (8.8)	28.6 (6.7)
**Foresta, 2005, (** 22) Italy, RCT	rhFSH 100	62	34 (5)	3.4 (2.2)	2.9 (1.5)	4.6 (1.2)	16.6 (9.0)*^c^*	15.9 (7.2)*^d^*	6.2 (3.4)*^d^*	29.8 (8.2)	25.3 (6.2)
	No tx	50	34 (5)	3.1 (2.1)	2.6 (1.2)	4.8 (1.4)	15.9 (8.3)*^c^*	16.5 (5.1)	6.8 (3.2)	28.9 (8.8)	26.2 (6.8)
**Selice, 2011**, (23) Italy, RCT	rhFSH 150	70	NR	NR	4.4 (1.7)	5 (1.8)	15.9 (5.4)	10.6 (10.6)	4.0 (4.2)	13.7 (6.9)	20.8 (15.4)
	Placebo	35	NR	NR	4.1 (1.5)	4.9 (2)	16.1 (5.8)	10.6 (11.4)	3.8 (4.0)	13.3 (6.4)	20.1 (16.3)
**Paradisi, 2014**, (24) Italy, NRCS	rhFSH 300	45	NR	NR	3.6 (1.3)	3.9 (1.4)	16.6 (5.5)*^c^*	26.2 (18.7)	7.2 (3.6)	24.6 (7.9)	27.0 (13.7)*^e^*
	Placebo	15	NR	NR	3.8 (1.4)	4.3 (1.8)	17.3 (5.9)*^c^*	25.0 (22.2)	7.4 (4.1)	27.6 (9.4)	27.7 (15.1)*^e^*
**Ding—Trial 1, (** 26) **2015**, China, RCT	rhFSH 50	36	33 (3)*^e^*	3.4 (3.5)*^f^*	3.4 (1.1)*^f^*	4.8 (1.9)*^f^*	13.2 (4.7)*^f^*	13.3 (5.6)	4.3 (2.1)	29.4 (6.2)	25.4 (5.1)
	rhFSH 100	38						16.2 (6.2)	5.2 (4.3)	30.7 (3.2)	24.8 (5.1)
	rhFSH 200	41						15.8 (7.7)	4.8 (5.2)	29.7 (9.2)	24.8 (10.1)
	rhFSH 300	40						14.1 (5.7)	5.1 (3.3)	31.7 (9.2)	23.8 (10.1)
	Placebo	30	36 (4)*^e^*	3.3 (2.2)*^f^*	3.1 (1.6)*^f^*	4.9 (1.5)*^f^*	12.2 (2.6)*^f^*	12.1 (4.1)	4.1 (3.3)	29.7 (9.2)	25.8 (9.1)
**Ding—Trial 2, (** 26) **2015**, China, RCT	rhFSH 300	40	33 (3)*^e^*	3.4 (3.5)*^f^*	3.4 (1.1)*^f^*	4.8 (1.9)*^f^*	13.2 (4.7)*^f^*	14.7 (5.2)	3.7 (3.2)	26.6 (6.2)	23.5 (8.3)
	Placebo	29	36 (4)*^e^*	3.3 (2.2)*^f^*	3.1 (1.6)*^f^*	4.9 (1.5)*^f^*	12.2 (2.6)*^f^*	NR	NR	NR	NR
***hCG and hMG (n* = *1 study)***											
Knuth, 1987, (27) Germany, RCT	hCG 2500 +hMG 150	17	31 (4)	5 (3.4)	4.6 (0.6)	3.6 (0.6)	12.4 (1.4)	NR	3.3 (0.8)*^g^*	32.1 (6.0)*^g^*	29.2 (6.0)*^g^*
	Placebo	20	33 (7)	6 (3.7)	5.2 (0.6)	4.2 (0.5)	12.9 (0.8)	NR	3.7 (0.7)*^g^*	37.1 (4.4)*^g^*	32.3 (4.2)*^g^*
**SERMs (n = 1 study)**											
***Tamoxifen (n* = *1 study)***											
AinMelk, 1987, (28) Canada, crossover RCT	Tamoxifen 20	16	Range 23-34	NR	7.5 (0.7)*^g^*	5.6 (0.9)*^g^*	21.2 (1.7)*^c,g^*	17.1 (3.1)*^g^*	NR	62.5 (14.2)*^g^*	32.5 (3.2)*^g^*
	Placebo										

There were no studies in the included sample that assessed the effectiveness of aromatase inhibitors for men with idiopathic infertility. All results are reported as mean (SD).

Abbreviations: hMG, human menopausal gonadotropin; hCG, human chorionic gonadotropin; NR, not reported; NRCS, nonrandomized controlled trial; RCT, randomized controlled trial; rhFSH, recombinant human FSH; SERM, selective estrogen receptor modulator; tx, treatment; uFSH-HP, urinary human FSH highly purified.

^*a*^Defined as progressive motility for all FSH studies, total motility (ie, could include motile, nonforward moving) for non-FSH studies.

^*b*^Study reports hormonal data for sample overall and does not report arm-level data.

^*c*^Reported testosterone value converted to universal units using https://unitslab.com/node/136.

^*d*^Study reports semen data for intervention responders and non-responders separately and have been reconstructed here for an overall intervention mean and SD.

^*e*^Progressive motility calculated from other motility parameters.

^*f*^Study reports hormonal and semen data for 3 trials (n = 354 patients) even though only 2 of the trials are relevant and included in this review (n = 254 total). The baseline data included in this table represent the mean and SD for the “treated group” (n = 272) and “placebo control group” (n = 82) across all 3 trials.

^*g*^Study reported mean and standard error of the mean (SEM) that was too large. A decision was made to interpret the value as an SD.

### Risk of bias in included studies

Overall, the methodological quality of the included studies was low or very low, and the quality of reporting was generally poor (Table 3). Of the 9 studies, 8 were described as randomized trials; however, only 2 provided sufficient information on the method of randomization and allocation concealment to judge whether these methods were adequate (22, 23). The remaining 6 RCTs did not report any explicit information on the randomization process or concealment methods, resulting in a judgment of high risk of bias (20, 21, 26-28). The NRCS, in which, by definition, the assignment of interventions was neither randomized nor concealed, also carried a high risk of bias (24). Because of limited reporting, the risk of bias related to blinding of participants, health care providers, and outcome assessors was difficult to assess in most studies. Consequently, our assessments were based on the absence of indications of bias, rather than on affirmative evidence that blinding was adequately implemented. We judged blinding of all 3 groups, patients, providers, outcome assessors, to be probably in place in 5 placebo-controlled studies (20, 26-28) and accordingly the risk of bias in these domains was assessed as *probably low*. In 3 studies that used a “no treatment’ control, blinding of participants and care providers was not feasible and was, therefore, rated as *high risk* of bias (21-23).

**Table 3:** Risk of bias of included studies (Reviews 1 and 2)

Author, Year, design	Item 1: Random sequence	Item 2: Allocation concealment	Item 3: Blinding of participants	Item 4: Blinding of providers	Item 5: Blinding of outcome assessors	Item 6: Outcome data not included
**Gonadotropins (n = 8 studies)** *^a^*						
***FSH (n* = *7 studies)*** *^a^*						
**Foresta, 1998, RCT (20)**	High	High	Probably low	Probably low	Probably low	Probably high
**Foresta, 2002, RCT (21)**	High	High	High	High	Low	Probably high
**Foresta, 2005, RCT (22)**	Low	Low	High	High	Low	Probably high
**Selice, 2011, RCT (23)**	Low	Low	High	High	Probably high	Probably high
**Paradisi, 2014, NRCS (24)**	High	High	High	High	Probably high	Probably high
**Ding, 2015a, RCT (26)**	High	High	Probably low	Probably low	Probably low	Low
**Ding, 2015b, RCT (26)**	High	High	Probably low	Probably low	Probably low	Low
***hMG + hCG (n* = *1 study)***						
**Knuth, 1987, RCT (27)**	High	High	Probably low	Probably low	Probably low	Probably low
**SERMs (n = 1 study)**						
***Tamoxifen (n* = *1 study)***						
**AinMelk, 1987, RCT (28)**	High	High	Probably low	Probably low	Probably low	High

There were no studies in the included sample that assessed the effectiveness of aromatase inhibitors for men with idiopathic infertility. Red cells were coded as definitely high bias, green cells were coded as definitely low risk of bias, yellow cells were coded uncertain (probably low or probably high).

Abbreviations: hCG, human chorionic gonadotropin; hMG, human menopausal gonadotropin; SERM, selective oestrogen receptor modulator.

^*a*^Quality appraisal for Trials 1 and 2 as methodological details were reported sparsely and on the whole for the 3 studies together.

Two of these studies clearly reported having a masked outcome assessor and were assessed as *definitely low risk* of bias in that domain (21, 22), whereas the other study provided no evidence of outcome assessor blinding and was judged as *probably high risk* of bias (23). In the NRCS, blinding of participants and health care providers was definitely not implemented, leading to a judgment of *definitely high risk* of bias. Similarly, the masking of outcome assessors was probably not in place, resulting in a rating of *probably high risk* of bias (24).

For most of included studies, outcome data were analyzed only for participants who completed the study, with limited or no reporting on dropouts or missing data. This lack of transparency made it difficult to assess risk of bias because of incomplete outcome data in analysis (attrition bias). One study, which reported minimal dropout (<5%), was assessed as having a *low risk* of attrition bias (26). Another study that reported “excellent” compliance with scheduled follow-up examinations attrition was assessed to have a *probably low risk* of bias (27). In contrast, 1 study that reported >15% dropouts without providing the numbers per study arm or reasons for dropout was judged to have a *high risk* of bias (28). Another study showed apparently unbalanced dropouts between study arms and was assessed to have a *probably high risk* of bias (22). Four studies did not report information on attrition, resulting in a judgment of *probably high risk* of bias (20, 21, 23, 24).

Overall, none of the included studies was rated as low risk in all bias domains, indicating that the methodological quality of the included studies was generally low or very low.

### Synthesis results

#### Review 1

Study level results for all sperm outcomes are presented in Table 4.

**Table 4:** Study results

				Semen parameters			
Author, Year, country, design	Arm	N analyzed	Months	Count (× 10^6^ sperm)	Concentration (× 10^6^/mL)	Morphology (% normal)	Motility (% forward)
**Gonadotropins (n = 7 studies)**							
***FSH (n* = *6 studies)***							
**Foresta, 1998**, (20) Italy, RCT	uFSH-HP 75	60	3	17.7 (9.6)	9.8 (7.3)	44.6 (4.8)	37.6 (5.9)
	Placebo	30	3	9.6 (3.3)	5.1 (2.9)	35.5 (5.4)	33.7 (5.9)
	MD (95% CI)			8.1 (5.4-10.8)	4.7 (2.6-6.8)	9.1 (6.6-11.4)	3.9 (1.3-6.5)
**Foresta, 2002**, (21) Italy, RCT	rhFSH 50	15	3	NR	5.8 (2.6)*^a^*	33.5 (9.3)*^a^*	32.2 (9.6)*^a^*
	rhFSH 100	15	3	NR	9.6 (3.6)*^a^*	43.2 (7.4)*^a^*	37.2 (8.8)*^a^*
	No tx	15	3	NR	5.6 (2.9)	36.8 (10.3)	27.6 (8.5)
	MD (95% CI)			NR	2.1 (0.1-4.1)*^a^*	1.6 (−4.6 to 7.8)*^a^*	7.1 (1.6-12.6)*^a^*
**Foresta, 2005 (** 22), Italy, RCT	rhFSH 100	62	3	30 (16.3)*^b^*	13.6 (7.5)*^b^*	NR*^c^*	NR*^c^*
	No tx	50	3	17.3 (6.6)	7.1 (3.5)	NR*^c^*	NR*^c^*
	MD (95% CI)			12.7 (8.2-17.2)	6.5 (4.4-8.6)	NR*^c^*	NR*^c^*
**Selice, 2011**, (23) Italy, RCT	rFSH 150	70	3	24.0 (28.1)	8.6 (11.3)	15.9 (8.4)	25.5 (17.7)
	Placebo	35	3	11.7 (11.9)	4.1 (4.2)	11.9 (6.0)	21.6 (16.9)
	MD (95% CI)			12.3 (4.63-20.0)	4.5 (1.5-7.5)	4.0 (1.2-6.8)	3.9 (−3.1 to 10.9)
**Paradisi, 2014**, (24) Italy, NRCS	rhFSH 300	45	4	64.1 (51.2)	19 (12.4)	26.5 (8.0)	27.0 (13.7)*^d^*
	Placebo	15	4	24.0 (15.3)	7.5 (4.6)	25.9 (8.0)	28.9 (14.4)
	MD (95% CI)			40.1 (23.3-56.9)	11.5 (7.2-15.8)	0.6 (−4.1 to 5.3)	−1.9 (−10.2 to 6.4)
**Ding, 2015a**, (26) China, RCT	rhFSH 50	36	3	16.9 (3.5)*^e^*	5.9 (3.9)*^e^*	28.2 (3.7)*^e^*	27.7 (4.7)*^e^*
	rhFSH 100	38	3	18.2 (5.7)*^e^*	6.9 (7.9)*^e^*	35.8 (9.7)*^e^*	28.7 (12.9)*^e^*
	rhFSH 200	41	3	29.7 (9.9)*^e^*	14.9 (7.2)*^e^*	35.8 (12.8)*^e^*	30.7 (7.1)*^e^*
	rhFSH 300	40	3	44.1 (12.4)*^e^*	19.1 (10.2)*^e^*	36.1 (12.8)*^e^*	29.7 (11.8)*^e^*
	Placebo	30	3	15.2 (3.9)	5.0 (2.3)	31.9 (10.4)	27.1 (3.2)
	MD (95% CI)*^d^*			12.4 (9.8-15.0)	10.0 (8.3-11.7)	2.2 (−1.9 to 6.3)	2.2 (0.3-4.1)
**Ding, 2015b**, (26) China, RCT	rhFSH 300	40	8	76.8 (25.6)	33.4 (13.2)	53.2 (14.1)	44.6 (11.5)
	Placebo	29	8	13.5 (4.5)	3.1 (2.1)	28.1 (11.1)	26.2 (10.2)
	MD (95% CI)			63.3 (55.2-71.4)	30.3 (26.1-34.5)	25.1 (19.2-31.1)	18.4 (13.3-23.5)
***hCG and hMG (n* = *1 study)***							
**Knuth, 1987**, (27) Germany, RCT	hCG 2500 +hMG 150	17	3	NR	3.8 (1.0)*^f^*	28.4 (5.2)*^f^*	35.0 (6.0)*^f^*
	Placebo	20	3	NR	4.8 (1.3)*^f^*	40.9 (3.6)*^f^*	36.4 (3.6)*^f^*
	MD (95% CI)			NR	−1.0 (−1.7 to −0.3)	−12.5 (−15.4 to −9.6)	−1.4 (−4.7 to 1.9)
**SERMs (n = 1 study)**							
***Tamoxifen (n* = *1 study)***							
**AinMelk, 1987**, (28) Canada, crossover RCT	Tamoxifen 20	16	6	17.7 (7.0)*^f^*	NR	58.8 (19.0)*^f^*	31.7 (10.2)*^f^*
	Placebo		6	18.4 (7.0)*^f^*	NR	59.6 (19.6)*^f^*	34.6 (11.2)*^f^*
	MD (95% CI)			−0.7 (−5.6 to 4.1)	NR	−0.8 (−14.2 to 12.5)	−2.9 (−10.3 to 4.5)

Abbreviations: hMG, human menopausal gonadotropin; hCG, human chorionic gonadotropin; MD, mean difference; NR, not reported; NRCS, nonrandomized controlled trial; RCT, randomized controlled trial; rhFSH, recombinant human FSH; SERM, selective estrogen receptor modulator; tx, treatment; uFSH-HP, urinary human FSH highly purified.

^*a*^Combined arms rhFSH 50 and 100

^*b*^Study reports semen data for intervention responders and nonresponders separately and have been reconstructed here for an overall intervention mean and SD

^*c*^Report “No significant variations were observed in other seminal parameters, such as forward motility and normal morphology, at the end of treatment in both groups (data not shown).”

^*d*^Study reported progressive motility in two categories; we combined the categories using a standard formula with an assumed correlation of 0.7.

^*e*^Combined arms rhFSH 50, 100, 200, and 300.

^*f*^Study reported mean and standard error of the mean (SEM) that was too large. A decision was made to interpret the value as an SD.

##### Total sperm count

**Figure 3:**
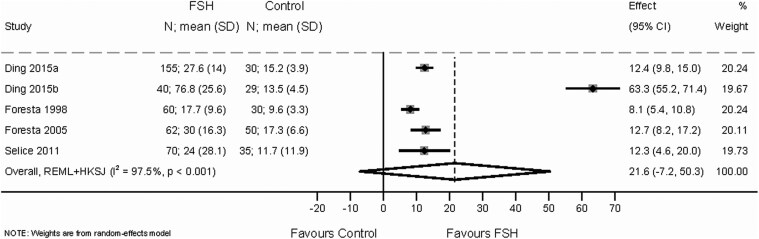
Meta-analysis of sperm count comparing FSH vs control.

**Figure 4:**
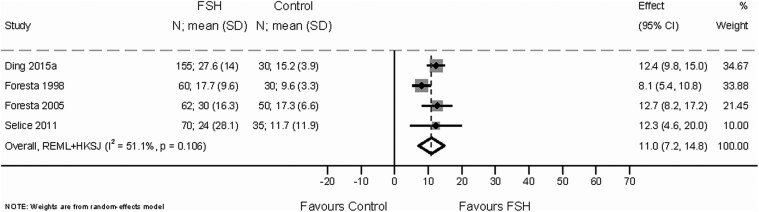
Sensitivity analysis of sperm count for FSH vs control, excluding Ding 2015b (26).

**Figure 5:**
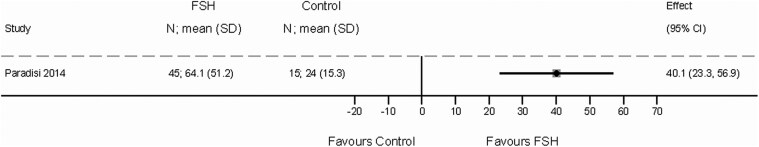
Summary results for sperm count comparing FSH vs control (nonrandomized comparison).

**Figure 6:**
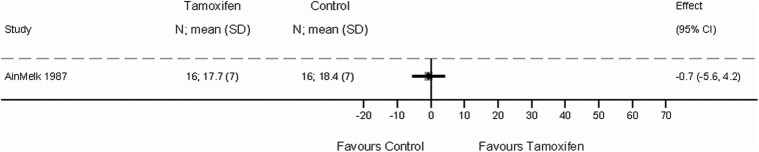
Summary results for sperm count comparing tamoxifen vs control.

Seven studies reported changes in sperm count following hormonal therapy. Six of these studies—comprising 5 RCTs (20, 22, 23, 26) and 1 NRCS (24)—evaluated FSH vs control in a total of 621 participants. One randomized crossover study with 16 participants evaluated tamoxifen vs control (28). The meta-analysis of FSH studies showed a nonsignificant MD of 21.6 × 10^6^ sperm (95% CI, −7.2 to 50.3) in favor of FSH; however, with substantial heterogeneity across studies (*I*^2^ = 97.5%; Fig. 3). A sensitivity analysis excluding the highly effective outlier study by Ding et al (2015b) (26) yielded a reduced but significant mean difference of 11.0 (95% CI, 7.2-14.8) with heterogeneity decreasing to *I*^2^ = 51.1% (see Fig. 4). Results from the NRCS also showed a significant difference favoring FSH (Fig. 5) (24). In contrast, the single study comparing tamoxifen vs control showed no evidence of a difference in sperm count between treatment groups (Fig. 6) (28).

##### Sperm concentration

**Figure 7:**
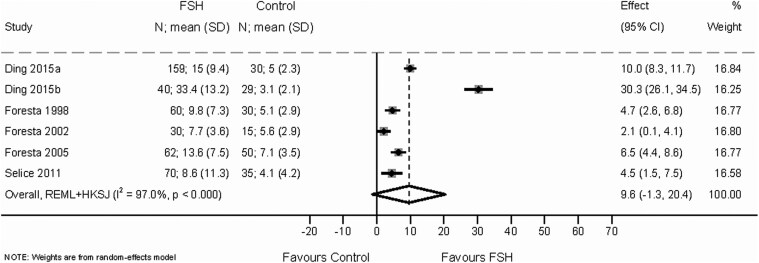
Meta-analysis of sperm concentration comparing FSH vs control.

**Figure 8:**
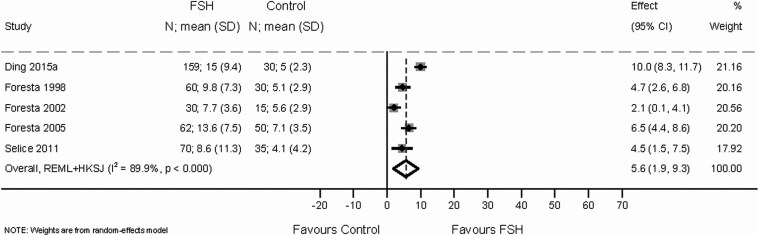
Sensitivity analysis of sperm concentration for FSH vs control, excluding Ding 2015b (26).

**Figure 9:**
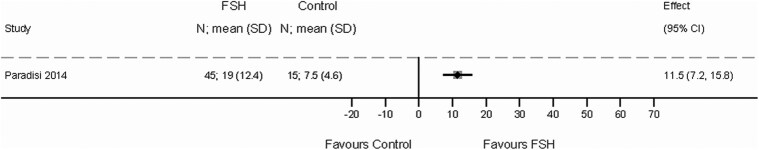
Summary results for sperm concentration comparing FSH vs control (nonrandomized comparison).

**Figure 10:**
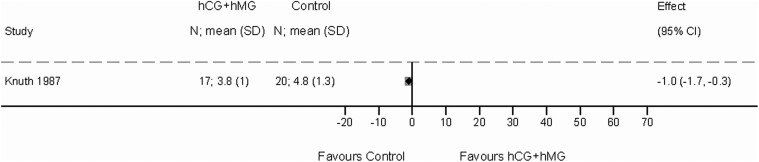
Summary results for sperm concentration comparing hCG + hMG vs control. Foresta 2005 could not be included in the forest plot as it did not report any numerical data, only “No significant variations were observed” (22).

Eight studies reported changes in sperm concentration following hormonal therapy. Seven studies—6 RCTs (20-23, 26) and 1 NRCS (24)—evaluated FSH vs control in 666 participants and 1 RCT evaluated hCG/hMG vs control in 37 participants. Overall, the meta-analysis results showed a nonsignificant difference in favor of FSH (MD 9.6 10^6^ sperm/mL; 95% CI, −1.3 to 20.4) but there was evidence of substantial heterogeneity across studies (*I*^2^ = 97.0%; Fig. 7). In line with the sperm count findings, a sensitivity analysis removing the outlier study by Ding et al (2015b) (26) showed a significant mean difference of 5.6 (95% CI, 1.9-9.3); although substantial heterogeneity was still present among studies (*I*^2^ = 89.9%; Fig. 8). Findings from the single NRCS favored FSH (Fig. 9) (24) In contrast, the results of the single RCT assessing hCG/hMG showed a significant difference in favor of the control group, suggesting a potential detrimental effect of the intervention (Fig. 10) (27).

##### Sperm morphology

**Figure 11:**
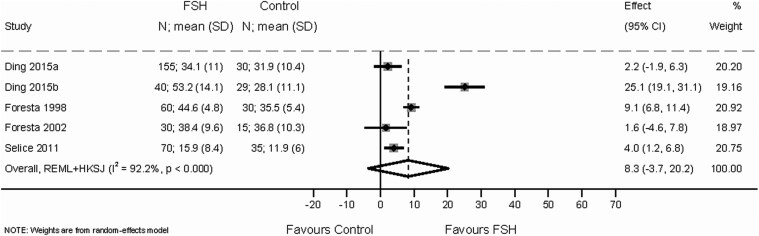
Meta-analysis of sperm morphology comparing FSH vs control. Foresta 2005 could not be included in the forest plot as it did not report any numerical data, only “No significant variations were observed” (22).

**Figure 12:**
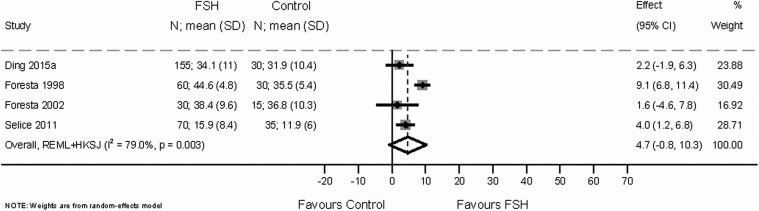
Sensitivity analysis of sperm morphology for FSH vs control, excluding Ding 2015b (26).

**Figure 13:**
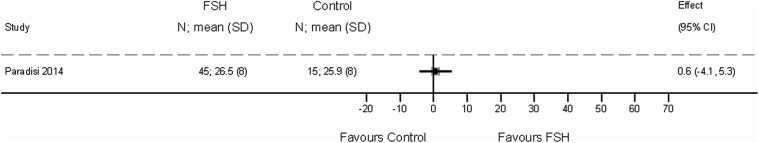
Summary results for sperm morphology comparing FSH vs control (nonrandomized comparison).

**Figure 14:**
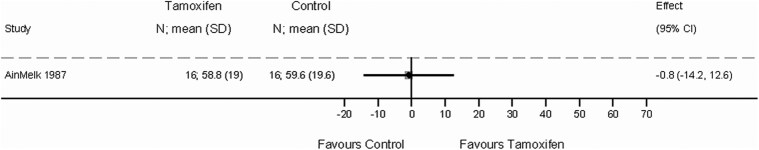
Summary results for sperm morphology comparing tamoxifen vs control.

**Figure 15:**
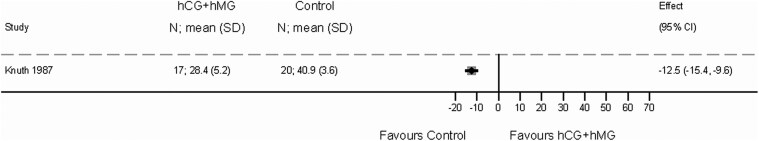
Summary results for sperm morphology comparing hCG + hMG vs control.

Nine studies reported changes in sperm morphology following hormonal therapy. These included 7 studies (6 RCTs (20-23, 26), 1 NRCS (24); n = 666) evaluating FSH vs control, 1 RCT evaluating hCG/hMG vs control (n = 37) (27), and 1 randomized crossover study evaluating tamoxifen vs control (n = 16) (28). Overall, the meta-analysis of the FSH studies showed a nonsignificant MD of 8.3% increase in normal morphology in favor of FSH (95% CI, −3.7 to 20.2), Fig. 11) with substantial heterogeneity among studies (*I*^2^ = 92.2%). A sensitivity analysis excluding the outlier study by Ding et al (2015b) (26) reduced both the effect estimate and the width of the confidence interval (MD 4.7%; 95% CI, −0.8 to 10.3); although heterogeneity was reduced, it remained substantial (*I*^2^ = 79.0%, Fig. 12). The NRCS showed no difference between treatment groups (Fig. 13) (24). Similarly, the RCT evaluating tamoxifen did not find a significant difference between treatment groups (Fig. 14) (28). In contrast, the RCT assessing hCG + hMG showed a significant improvement in sperm morphology favoring the control group (Fig. 15) (27).

##### Sperm motility

**Figure 16:**
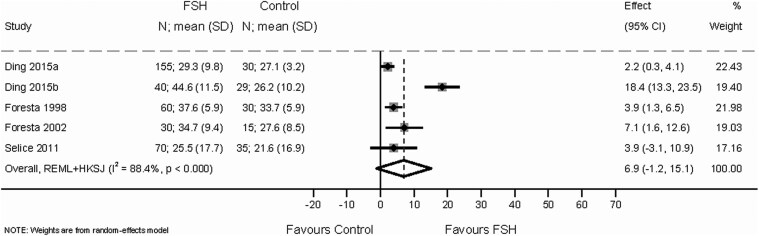
Meta-analysis of sperm motility comparing FSH vs control.

**Figure 17:**
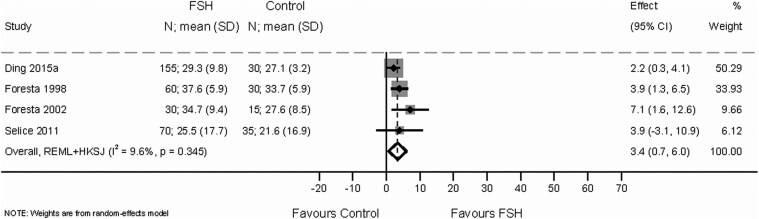
Sensitivity analysis of sperm motility for FSH vs control, excluding Ding 2015b (26).

**Figure 18:**
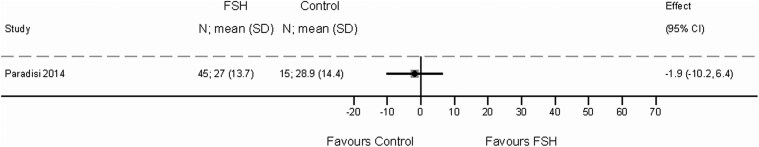
Summary results for sperm motility comparing FSH vs control (nonrandomized comparison).

**Figure 19:**
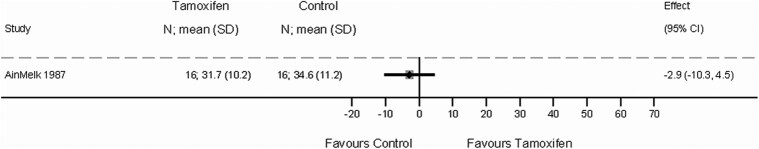
Summary results for sperm motility comparing tamoxifen vs control.

**Figure 20:**
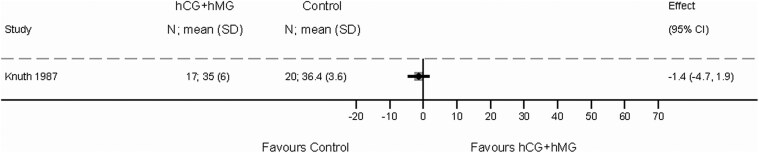
Summary results for sperm motility comparing hCG + hMG vs control.

Nine studies reported changes in sperm motility following hormonal therapy: 7 studies (6 RCTs (20-23, 26), 1 NRCS (24); total n = 666) evaluating FSH vs control, 1 RCT evaluating hCG/hMG vs control (n = 37) (27), and 1 randomized crossover study evaluating tamoxifen vs control (n = 16) (28). It is worth noting that all studies evaluating FSH reported “forward motility” (ie, % A + B motile sperm), whereas the non-FSH studies reported overall motility (ie, % A + B + C motile sperm). The meta-analysis of FSH studies demonstrated a nonsignificant mean difference of a 6.9% increase in forward motile sperm in favor of FSH (95% CI −1.2, 15.1). However, there was considerable heterogeneity across studies (*I*^2^ = 88.4%, Fig. 16). A sensitivity analysis excluding the outlier study by Ding et al (2015b) (26) yielded a significant mean difference of a 3.4% increase in forward motile sperm (95% CI, 0.7-6.0) with markedly reduced heterogeneity (*I*^2^ = 9.6%, Fig. 17). In contrast, the NRCS evaluating FSH as well as the 2 RCTs evaluating tamoxifen and hCG + hMG, respectively, showed no evidence of a treatment effect on sperm motility (Figs. 18-20) (24, 27, 28).

#### Review 2

##### Embryos and oocytes

We did not identify any studies reporting these outcomes.

##### Pregnancy

**Figure 21:**
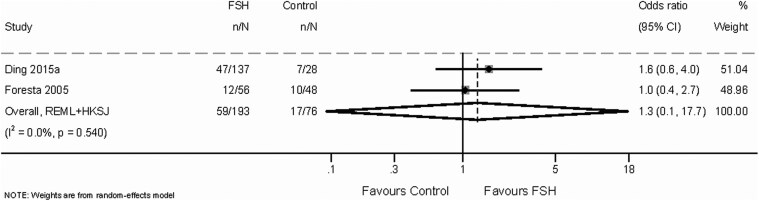
Meta-analysis of pregnancy outcome comparing FSH vs control.

Two RCTs reported pregnancy outcomes (generically defined) following FSH therapy (22, 26). Both studies showed numerically higher pregnancy rates in the FSH group than the control group, with a more pronounced effect observed in the study by Ding et al (2015a) (26). However, the effect sizes were small, the CIs were wide, and the overall estimate of effect was not statistically significant (OR 1.3; 95% CI, 0.1-17.7; Fig. 21).

## Discussion

Approximately one half of couples seeking fertility treatment is affected by male infertility (1, 2) Most men with male infertility are normogonadotropic and have no identified etiology. The high costs of IVF/ICSI have incentivized some clinicians to use hormonal therapies in an attempt to improve semen quality and other reproductive outcomes in affected couples; however, this practice remains unproven. We aimed to objectively appraise the effectiveness of hormonal treatments to improve reproductive outcomes in normogonadotropic men with abnormal semen parameters.

### Summary of the main results

Across several studies, FSH therapy was associated with small but consistent improvements in key semen parameters, including sperm count, concentration, and motility, compared with control or placebo. Meta-analyses initially showed variable results; however, after exclusion of an outlier study (28), the overall effect became more consistent and suggested modest, potentially clinically meaningful benefits in sperm count, concentration, and motility. Improvements in sperm morphology were minimal and remained uncertain. In contrast, treatments with hCG/hMG or tamoxifen did not show benefit, and in some cases were associated with worse semen outcomes compared to control.

Regarding pregnancy outcomes following assisted reproduction techniques, only 2 small studies reported relevant data (22, 26). Although both suggested a higher rate of pregnancy in the FSH groups, the pooled estimate did not demonstrate statistical significance. No distinction was given between clinical and ongoing pregnancies. The small sample size and wide CIs preclude firm conclusions on clinical efficacy for these outcomes.

### Strengths and limitations of the current evidence base

The current evidence base is subject to important limitations. The included studies were heterogeneous in their intervention protocols, particularly with respect to the dose and treatment regimens of FSH, which varied across trials. This clinical heterogeneity may have contributed to variability in treatment effects. Risk of bias was high or unclear in most studies because of inadequate reporting or concerns regarding the randomization process, allocation concealment, and blinding procedures. Attrition data were often insufficiently described. Some studies claimed 100% complete follow-up but failed to detail how many randomized participants were excluded from each study arm, the reasons for their exclusion, or whether any outcome data were missing. Moreover, there was also no indication of how missing data, if any, were handled statistically. This lack of transparency raised concerns, creating the impression of implausibly “clean” data with very few missing values, given the study context. However, given the generally small sample sizes of the included studies, it is possible that data were collected in highly controlled settings with rigorous and comprehensive follow-up of enrolled participants. If so, results may not accurately reflect real-world clinical practice or be easily generalizable.

Despite being published between 1987 and 2015, spanning the introduction and evolution of the CONSORT reporting standards (29), the overall quality of reporting was poor. The upcoming CONSORT 2025 update includes a new requirement to clearly define which participants are included in each analysis, their treatment allocation, and the handling of missing data (30). Future trials should rigorously follow the CONSORT guidelines to enhance transparency and reproducibility.

Four of the included studies were conducted in Italy by the same research group (20-23). Although 1 paper associated with this author team has been retracted, the reason for the retraction is unknown (31). We found no evidence suggesting a connection between the retracted paper and the studies included in this review. We also found no direct reason to question the validity of the four included studies, which, to our knowledge, appear to represent distinct trials and populations.

Publication bias could not be formally assessed due to the small number of studies, but the uniformly positive direction of findings raises concerns about selective reporting. The lack of long-term or clinically relevant outcomes, such as pregnancy and live birth or sustained improvements post-treatment, weakens the clinical applicability of our findings. Hormone treatments were limited to FSH, hCG/hMG, and tamoxifen. No eligible studies assessed aromatase inhibitors or clomiphene despite their off-label use.

### Interpretation of findings in the context of existing evidence

Previous systematic reviews have yielded similarly cautious conclusions regarding FSH treatment. A 2013 Cochrane review found some evidence supporting increased pregnancy and live birth rates with gonadotrophin treatment for men with idiopathic male factor subfertility, but the conclusions were hampered by small study sizes and heterogeneous populations (32). More recent systematic reviews echo our findings, indicating potential benefits for FSH in improving semen parameters, particularly sperm count and concentration, in normogonadotropic men with idiopathic oligozoospermia (33) or idiopathic OAT (34). In particular, it has been postulated that higher doses of FSH may be associated with greater effects on semen quality in men. We also note that the study with the largest effect size used high doses of FSH (300 U) (26). Future studies are required to determine whether potential effects of FSH on semen quality are dose-dependent. Additionally, increased pregnancy rates have been reported following FSH administration to the male partner in couples experiencing infertility (35). However, these systematic reviews also highlight ongoing uncertainty because of methodological issues, including heterogeneity in the populations of men with infertility, variability in treatment protocols across studies, and the limited number of studies and small sample sizes of available trials, often resulting in low-quality evidence (35). Notably, the present review did not find any new studies on this topic published after 2015 (33).

Of note, a nonsignificant worsening in semen parameters was observed in a study in which hCG was administered with hMG. hCG activates the LH receptor, which is needed for the high intratesticular levels of testosterone required for sperm maturation (36). It is therefore counterintuitive that hCG should paradoxically impair spermatogenesis. However, men abusing supraphysiological androgens have a prolonged suppression spermatogenesis through mechanisms which are partially testicular (37). It therefore cannot be excluded that hCG might plausibly impair spermatogenesis if administered to men without hypogonadism.

Published systematic reviews assessing clomiphene or tamoxifen (SERM) have produced mixed findings. Some showed improvements in sperm concentration and motility during clomiphene treatment compared with pretreatment levels (38), or placebo (39, 40), whereas others found the evidence base to be limited and too heterogeneous to evaluate the efficacy of SERM for treating male infertility (41-43). Similarly, prior reviews on aromatase inhibitors for male infertility were less convincing, concluding that the number and quality of available studies remain insufficient to support meaningful clinical benefit (44, 45).

Although current European and American guidelines provide weak or conditional recommendations for hormonal treatment in idiopathic male infertility, our findings add cautious support to the potential role of FSH in improving semen parameters in men. However, the clinical relevance of modest improvements in semen parameters remains uncertain, particularly given the lack of robust data on pregnancy and live birth outcomes.

It is also notable that the studies evaluating hCG/hMG and tamoxifen do not support their efficacy in this population.

### Strengths and limitations of the review process

The primary strength of this review is its rigorous and methodologically transparent approach. Search strategies were comprehensive, inclusion criteria were well-defined in collaboration with clinical experts, and risk of bias was assessed in line with recommended, validated tools. The use of random-effects meta-analyses with sensitivity checks strengthens confidence in the consistency of observed effects, particularly for FSH.

However, our findings are constrained by the limitations of the underlying studies and by the small number of eligible trials, especially for outcomes such as pregnancy and live birth.

### Implications for clinical practice

Taken together, our findings provide cautious support for the future potential of FSH monotherapy in normogonadotropic men with idiopathic oligo- or asthenozoospermia, particularly where semen quality is borderline, and assisted reproduction is being considered. However, clinicians and patients should be aware that the expected benefits are modest and short-term, with uncertain translation to pregnancy or live birth. More robust evidence from a large, well-designed RCT with live birth as the primary outcome is required before routine use of FSH for idiopathic male infertility can be recommended.

Given the absence of demonstrated benefit for hCG/hMG and tamoxifen, and the lack of evidence for other hormonal agents, current clinical practice should not extend to these treatments without further substantiation.

### Future research directions

To advance the field, future research should prioritize robust methodology with full adherence to CONSORT guidelines, including transparent reporting of randomization, blinding, attrition, and missing data. Studies should focus on large, multicenter trials with sufficient statistical power to evaluate both intermediate outcomes, such as semen parameters, and clinically meaningful endpoints, including pregnancy and live birth. In addition, greater transparency in reporting is essential, particularly with respect to missing data, funding sources, and potential conflicts of interest, which were inadequately reported in the existing evidence base.

## Data Availability

This is an evidence synthesis; quantitative data are presented in the text or contained within tables, figures, and appendices. No new data, suitable for data sharing, have been created in the preparation of this synthesis. All queries should be submitted to the corresponding author for consideration.

## Abbreviations

hCG: human chorionic gonadotropin
hMG: human menopausal gonadotropin
ICSI: intra-cytoplasmic sperm injection
IVF: in vitro fertilization
MD: mean difference
NRCS: nonrandomized controlled study
OAT: oligo-astheno-teratozoospermia syndrome
OR: odds ratio
RCT: randomized controlled trial
rhFSH: recombinant human FSH
SERM: selective estrogen receptor modulator
uFSH: urinary human FSH
uhFSH-HP: urinary human FSH highly purified
